# Analysis of fungal diversity in the feces of *Arborophila rufipectus*

**DOI:** 10.3389/fvets.2024.1430518

**Published:** 2024-10-14

**Authors:** Xiaoping Ma, Junshu Li, Zhiguo Li, Benping Chen, Zhenwen Ling, Shenglin Feng, Zhijun Zhong, Guangneng Peng, Ya Wang, Yaozhang Jiang, Yu Gu

**Affiliations:** ^1^Key Laboratory of Animal Disease and Human Health of Sichuan Province, College of Veterinary Medicine, Sichuan Agricultural University, Chengdu, China; ^2^Sichuan Laojunshan National Nature Reserve, Yibin, China; ^3^Bioengineering Department, Sichuan Water Conservancy Vocational College, Chengdu, China; ^4^College of Life Sciences, Sichuan Agricultural University, Chengdu, China

**Keywords:** *Arborophila rufipectus*, intestinal fungus, high-throughput sequencing, feces, endangered species, potential pathogens, ITS rRNA

## Abstract

**Background:**

Intestinal fungal composition plays a crucial role in modulating host health, and thus is of great significance in the conservation of endangered bird species. However, research on gut fungal composition in birds is limited. Therefore, in this study, we aimed to examine gut fungal community and potential fecal pathogen composition in wild *Arborophila rufipectus*.

**Methods:**

Fecal samples were collected from the habitats of wild *A. rufipectus* and *Lophura nycthemera* (a widely distributed species belonging to the same family as *A. rufipectus*) in summer and autumn. Thereafter, RNA was collected and the internal transcribed spacer rRNA gene was sequenced via high-throughput sequencing to investigate seasonal variations in intestinal core fungi, microbial fungi, and potential pathogenic fungi.

**Results:**

The gut microbiota of *A. rufipectus* and *L. nycthemera* were highly similar and mainly consisted of three phyla, Ascomycota (58.46%), Basidiomycota (28.80%), and Zygomycota (3.56%), which accounted for 90.82% of the fungal community in all the samples. Further, the predominant genera were *Ascomycota_unclassified* (12.24%), *Fungi_unclassified* (8.37%), *Davidiella* (5.18%), *Helotiales_unclassified* (2.76%), *Wickerhamomyces* (1.84%), and *Pleosporales_unclassified* (1.14%), and the potential fecal pathogens identified included *Candida*, *Cryptococcus*, *Trichosporon*, and *Malassezia*.

**Conclusion:**

Our results provide evidence that the diversity of intestinal fungi in the endangered species, *A. rufipectus*, is similar to that in the common species, *L. nycthemera*, and may serve as a basis for monitoring the status of *A. rufipectus* and for developing conservation measures.

## 1 Introduction

*Arborophila rufipectus* (family, Phasianidae; order, Galliformes), a medium-sized mountain partridge with rich colors, is a national key protected wild bird (IUCN, 2016) that is endemic to China. Presently, the species is scattered around Southwest China, including Sichuan and Yunnan provinces (1), and its population in reserves is estimated at 2200 individuals (2). Further, given that the bird nests on the ground and has weak flight ability, it is highly vulnerable to natural predators (3), and owing to its small population and limited distribution, its habitat is scattered and isolated (4).

The intestinal tract provides a suitable environment for the growth and multiplication of various microbial communities, including bacteria, fungi, and archaea (5, 6), and together with these numerous microbes, form a complex and diverse ecosystem (7, 8). Under normal circumstances, the microorganisms in the gut can help the host perform essential functions, such as nutrient metabolism and immune enhancement (9–11). However, when this community is unbalanced or host immunity is weakened, these microbes can cause various diseases (12). Relative to bacteria, fungi account for a small proportion (approximately 0.1%) of the gut microbiota (8, 13, 14). Therefore, most studies on the gut microbiota have been focused on identifying and analyzing intestinal bacteria, while the importance of intestinal fungi has been largely ignored (15). Studying digestive tract fungal composition in this species may help clarify its dietary and possible migratory routes (2). Notably, fungi possess their own unique metabolic pathways (16), and imbalance in the intricate relationship between fungi and bacteria in the gut can result in health complications (17, 18).

Existing studies on the intestinal microflora of birds have been predominantly focused on chickens (19), while research on wild birds, particularly, endangered bird species, is limited possibly owing to the strong ability of most birds to migrate and fly, making data collection to study their gut microbiota characteristics challenging (19). Therefore, further studies, especially on the composition of fungal communities in the gut of wild birds, are needed. Therefore, in this study, we aimed to assess the diversity of gut fungi, including potential pathogenic fungi, in feces from wild *A. rufipectus*. For comparison, we also studied the diversity of gut fungi in *Lophura nycthemera*, which belongs to the same family as *A. rufipectus* and is also characterized by a weak flying ability and has feeding habits similar to that of *A. rufipectus*. However, unlike *A. rufipectus*, which is an endangered species, *L. nycthemera* is widely distributed and abundant in the Sichuan Laojunshan Mountain National Nature Reserve. The results of this study may improve understanding regarding the dominant fungi in the gut of *A. rufipectus*, and may promote efforts aimed at protecting this wild bird species.

## 2 Materials and methods

### 2.1 Sampling

Samples were collected from the Sichuan Laojunshan Mountain National Nature Reserve, China (103°57′–104°04′E, 28°39′–28°43′N). In brief, 152 fecal samples, 23 and 129 of which were for *L. nycthemera* and *A. rufipectus*, respectively, were collected from the abovementioned sampling site. In total, 10 samples each for *L. nycthemera* (collected in summer or June, A1-A10), *A. rufipectus* (collected in summer, B1-B10), and *A. rufipectus* (collected in autumn or September, C1-C10) were selected for high-throughput sequencing according to time of year and fecal quality. The samples were collected using separate tools and contact with the soil was avoided to minimize contamination. The distance between the different sample collection points was at least 5 m. Further, the samples were collected by a professional forest ranger based on the morphological characteristics of the feces of *A. rufipectus* and *L. nycthemera* (Supplementary Figures S1, S2). After collection, the samples were stored in a sterile centrifuge tube and temporarily placed in a portable freezer at −20°C and transported to the laboratory. At the laboratory, some of the samples were placed in liquid nitrogen for processing, while others were preserved in 15% glycerol for subsequent culture isolation. The range of the gut microbial characteristics of *L. nycthemera* expands in autumn owing to its dietary and migratory habits during this period; therefore, fecal samples were not found in the demarcated area during this period (20).

### 2.2 Isolation and identification of fungi

Each of the samples was inoculated onto potato dextrose agar and Sabouraud dextrose agar using disposable sterile inoculation rings. Thereafter, whole plates were cultured in a mold incubator at 25°C and fungal growth was observed for 12 h. Fungal colonies of different shapes and colors were selected and streaked for isolation until unique pure colonies were obtained. Lysis buffer for microorganisms and direct polymerase chain reaction (PCR; 9,164; Takara, Beijing, China) were added for fungal DNA extraction. PCR amplification of the internal transcribed spacer 2 (ITS2) region (21) of the fungal DNA was performed using universal primers: ITS1 (5′-GTGARTCATCGAATCTTTG-3′) and ITS2 (5′-TCCTCCGCTTATTGATATGC-3′). Thereafter, ITS gene sequencing was performed for fungal species identification. The PCR mixture consisted of 2.0 μL each of the forward and reverse primers, 2.0 μL of DNA template, and 9.0 μL of EmeraldAmp MAX PCR Master Mix (RR320Q; Takara, Beijing, China), with ddH_2_O added to obtain a total volume of 25 μL. The cycle conditions for PCR were as follows: 10 min initial denaturation at 94°C followed by 32 cycles of 30 s at 94°C, 30 s at 55°C, and 1 min at 72°C, and final extension for 10 min at 72°C. Further, the sequencing of the PCR products was performed by Sangon Biotech (Shanghai, China). Subsequently, we analyzed the sequencing data using NCBI BLAST. Sequences were searched against the NCBI GenBank and UNITE databases, and phylogenies were constructed using MEGA5 based on the obtained sequence data (20).

### 2.3 DNA extraction

DNA was extracted from the fecal samples using the cetyltrimethylammonium bromide method, with nuclease-free water as the blank control. After elution for PCR analysis with 50 μL of elution buffer, whole DNA was collected, stored at −80°C, and transported to LC-Biotechnology Co., Ltd. (Hang Zhou, China) for analysis.

### 2.4 PCR amplification and ITS rRNA sequencing

Specific primers (F: 5′-GAACCWGCGGARGGATCA-3′, R: 5′-GCTGCGTTCTTCATCGATGC-3′) were used to amplify the ITS1 region of the ITS rRNA gene sequence (22). The PCR reaction was performed using 2.5 μL of the reverse and forward primers, 12.5 μL of Phusion Hot Start Flex 2X Master Mix (M0536S; NEB, Beijing, China), 25 ng of template DNA, and PCR-grade water was added to make a final volume of 25 μL. The PCR conditions were as follows: 30 s initial denaturation at 98°C followed by 32 cycles of 10 s at 98°C, 30 s at 54°C, and 45 s at 72°C, and then final extension for 10 min at 72°C. Subsequently, 2% agarose gel electrophoresis was performed to verify the PCR products. AMPure XT beads were used for purification (Beckman Coulter Genomics, Danvers, MA, United States), while Qubit was used for quantification (Invitrogen, Waltham, MA, United States). Further, the Illumina Library Quantification Kit (Kapa Biosystems, Woburn, MA, United States) was used to prepare the amplicon pool for sequencing. The amplicon library number and size were assessed using an Agilent 2,100 Bioanalyzer (Agilent, Santa Clara, CA, United States) and sequenced on a NovaSeq PE250 platform (Illumina, San Diego, CA, United States) as reported previously (20).

### 2.5 Data analysis

The samples were sequenced using the Illumina NovaSeq platform in accordance with the recommendations of LC-Biotechnology Co., Ltd. Paired-end reads were assigned to the samples based on their unique barcodes. Thereafter, the samples were truncated by removing the barcode and primer sequences, and paired reads were assembled using PEAR, followed by quality filtering using fqtrim (v0.94) to obtain high-quality clean labels. Quality filtering was further performed using vSearch (v2.3.4) to screen chimeric sequences, and DADA2 used to obtain feature tables and sequences (20, 23, 24). Further, the QIIME2 algorithm was used to determine alpha and beta diversity indices. The same number of sequences was randomly extracted by reducing the number of sequences to the minimum and relative abundances (X fungal count/total count) were compared. Venn diagrams and box plots showing the results of the *α* diversity analysis and principal coordinate and cluster analyses diagrams showing the results of *β* diversity analysis, and stacked bar diagrams and heat maps showing the results of species analysis were generated using R software v3.5.2 (R Core Development Team, Vienna, Austria). The ribosomal database project and UNITE databases were used for species classification and subsequent analyses to ensure complete and accurate annotation results (annotation threshold: confidence greater than 0.7). Linear discriminant analysis (LDA) effect size (LEfSe) analysis was performed using OmicStudio tools [^1^(25) and OmicStudio Analysis (v1.0)].^2^

## 3 Results

### 3.1 Sequencing data analysis

**Figure 1:**
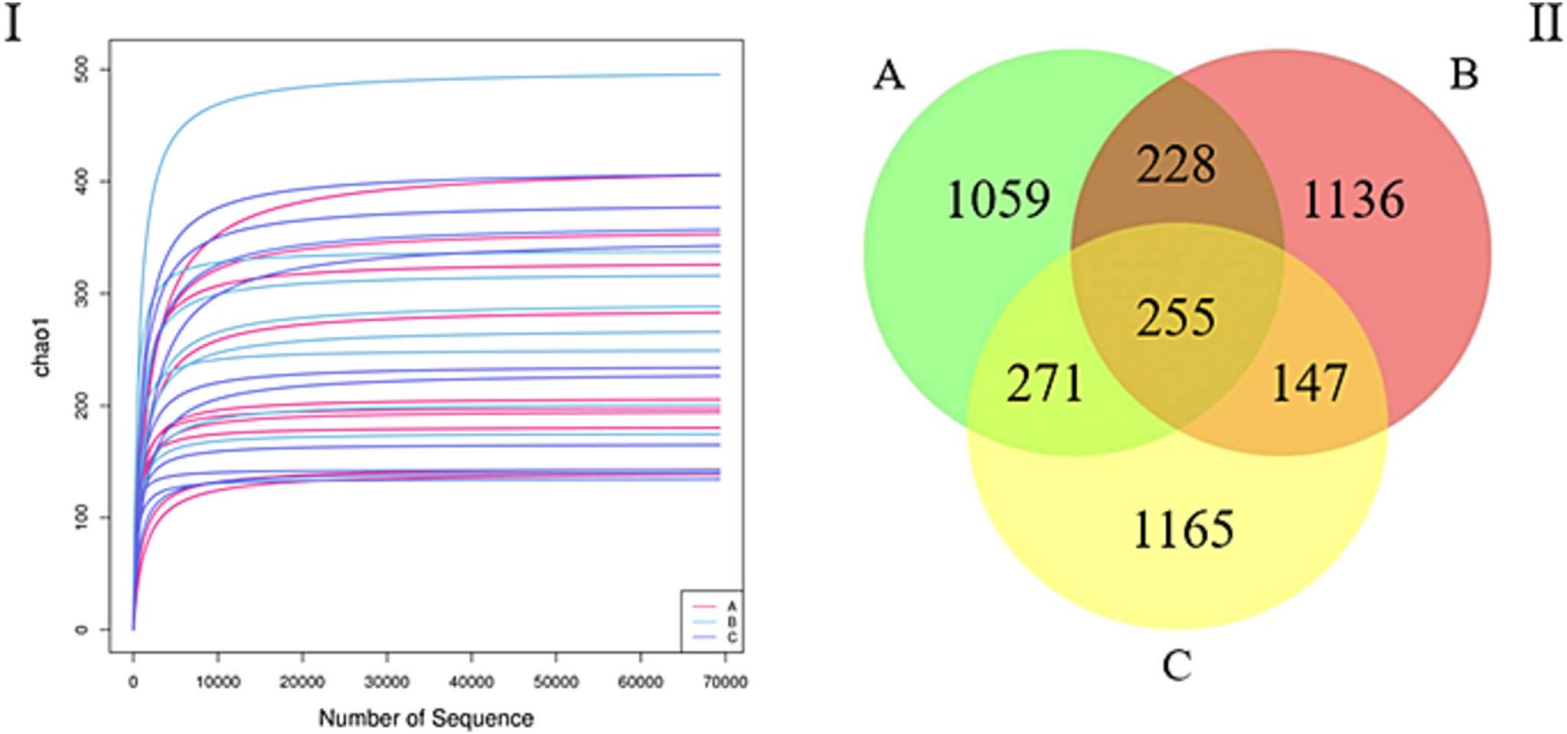
(I) Rarefaction curve. The curves with assorted colors show the observed operational taxonomic units (OTUs; *y*-axis) for each sample at different number of sequences (*x*-axis). The flat curves indicate that the sequencing depth sufficiently reflects most of the microbial data in the samples. (II) Venn diagram. OTU distribution in the three groups.

In total, 3,462,543 sequences were obtained after quality control. The mean number of sequences for all the samples was 115,418. The sequencing quality curves were flat and the number of operational taxonomic units (OTUs) was close to saturation, indicating that the sequencing depth sufficiently reflects most of the microbial data in the samples (Figure 1I). Further, via cluster analysis, we identified 1,813 (33.47%), 1,766 (32.60%), and 1,838 (33.93%) OTUs for samples A, B, and C, respectively, and the number of unique OTUs in the A, B, and C groups were 1,059, 1,136, and 1,165, respectively, accounting for 19.55, 20.97, and 21.50% of the total OTUs, respectively, while the number of OTUs common to all three groups was 225, accounting for 4.70% of the total number of OTUs (Figure 1II).

### 3.2 Analyses of OTU abundance and diversity indexes

**Figure 2:**
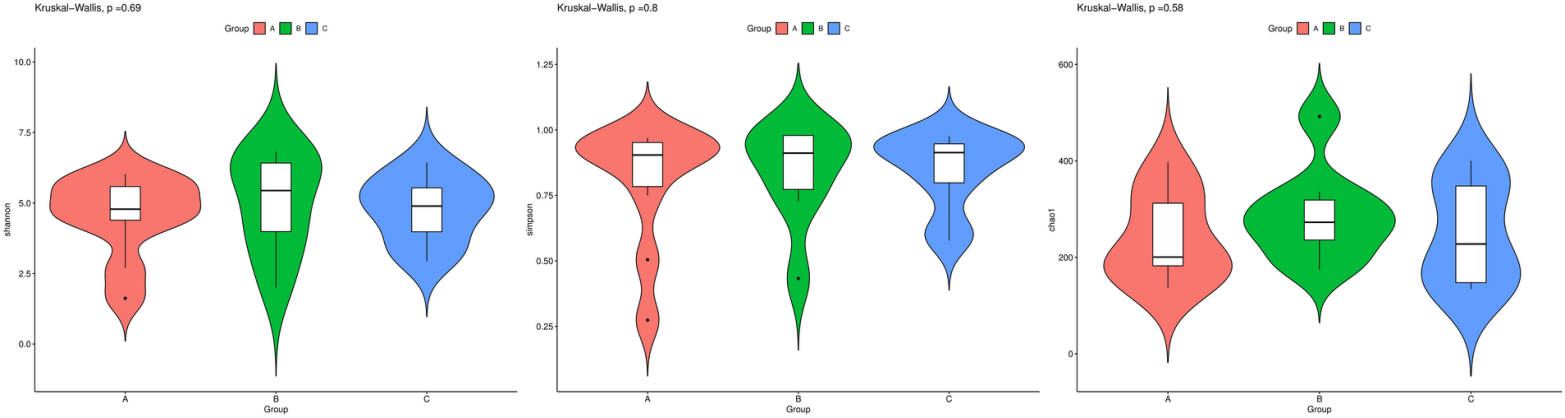
Fungal species richness in the guts of wild *Arborophila rufipectus* and *Lophura nycthemera* determined via internal transcribed spacer rRNA sequencing (I). Comparison of Shannon, Simpson, and Chao1 indices between groups B and C. (II) Comparison of Shannon, Simpson, and Chao1 indices between groups A and B. (III) Comparison of Shannon, Simpson, and Chao1 indices between groups A, B, and C.

Various parameters, including Shannon, Simpson, and Chao1 indices, were used to estimate the *α* diversity of the fungal community in the gut of the test birds (Figure 2). The results obtained for other parameters are presented in Supplementary Table S1. The Chao1 index was used to estimate the number of species in the community, while the Shannon index was used to estimate diversity, with a higher value indicating greater uncertainty (or higher diversity). The Simpson index, ranging between 0 and 1, with values closer to 1 indicative of a high level of species richness and uniformity in the community, was used to estimate species richness and uniformity. Our results showed no significant differences among the three groups with respect to *α* diversity (*p* > 0.05).

**Figure 3:**
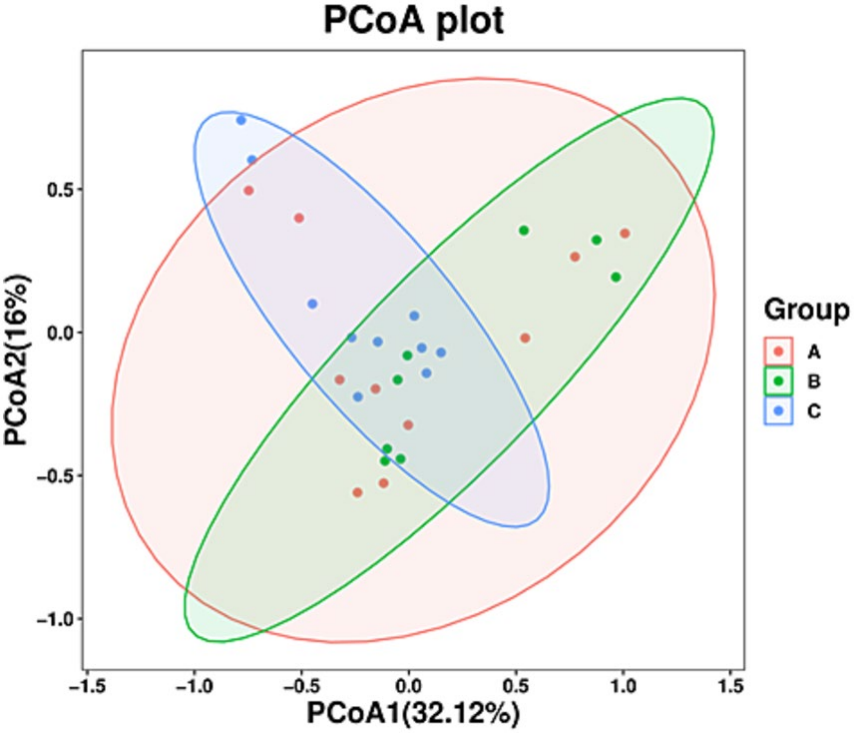
Principal coordinate analysis of fungal communities in the guts of wild *Arborophila rufipectus* and *Lophura nycthemera*. The dots represent samples, and distinct colors represent distinct groups (*p* > 0.1).

Principal coordinate analysis showed no clustering for the fecal samples in the three groups (Figure 3). An unweighted UniFrac distance matrix was obtained and in our clustering analysis, different colors represented different groups, and a smaller distance between samples was indicative of a greater level of similarity in terms of microbial composition and structure (i.e., smaller differences). The results obtained showed that three groups were not separated by large distances based on differences in time of sample collection and species, and substantial overlap was observed between the three groups. This observation indicated that the differences with respect to sampling time and species were small, possibly owing to the fact that *A. rufipectus* and *L. nycthemera* belong to the same family, Phasianidae. However, the time difference between sampling for groups B and C was only 3 months. These results were further supported by clustering analysis based on the unweighted pair group method with arithmetic mean (UPGMA; Supplementary Figure S3).

Further, *α* diversity analysis showed similarities in the diversity of intestinal fungi across the different seasons and species (*p* > 0.05). These results indicated that seasonal changes do not affect the diversity and richness of fungal communities in the intestinal tract of wild *A. rufipectus*.

### 3.3 Analysis of community composition

**Figure 4:**
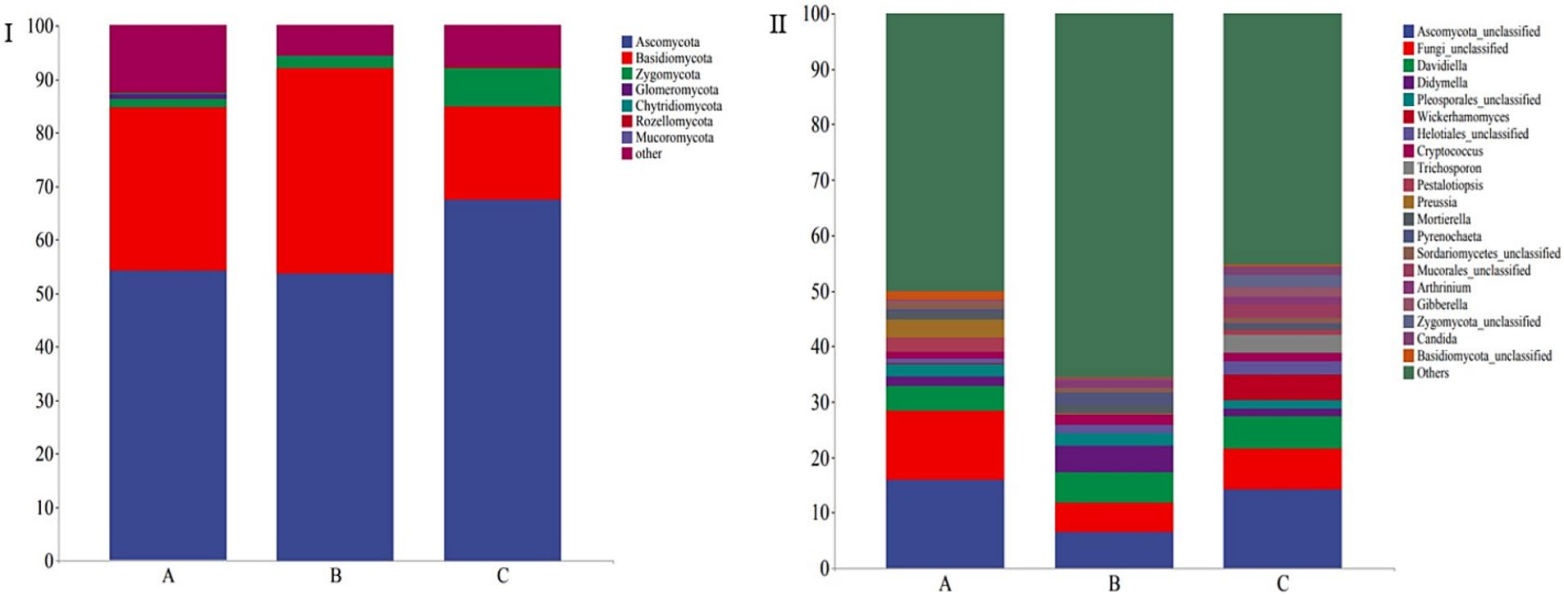
Stacked bar plots showing the average relative abundances of gut fungi in wild *Arborophila rufipectus* and *Lophura nycthemera* at the (I) phylum and (II) genus levels.

The taxonomic composition of 30 fecal samples was analyzed, and mean relative abundances at the phylum and genus levels were determined. The dominant phyla were Ascomycota (58.46%), Basidiomycota (28.80%), and Zygomycota (3.56%), accounting for 90.82% of the total fungal community in the samples. Further, the five dominant genera included *Ascomycota_unclassified* (12.24%), *Fungi_unclassified* (8.37%), *Davidiella* (5.18%), *Helotiales_unclassified* (2.76%), and *Wickerhamomyces* (1.84%), and the top seven core genera constituted 30.60% of all the fungi identified in all the samples (Figure 4). Ascomycota, Basidiomycota, and Zygomycota also constituted the dominant phyla in all the groups, and their relative abundances (>1%) were 54.28, 53.65, and 1.92%, respectively, in group A, 30.45, 38.48, and 17.48% respectively, in group B, and 1.63, 1.92, and 7.13%, respectively, in group C (Figure 4I).

At the genus level, *Ascomycota_unclassified* (16.00%), *Fungi_unclassified* (12.33%), *Davidiella* (4.49%), *Didymella* (4.53%), *Pleosporales_unclassified* (2.59%), *Wickerhamomyces* (2.15%), *Helotiales_unclassified* (1.77%), *Cryptococcus* (1.42%), *Trichosporon* (1.29%), and *Pestalotiopsis* (1.22%) were the dominant genera (>1%) in group A; *Ascomycota_unclassified* (6.52%), *Fungi_unclassified* (5.36%), *Davidiella* (5.35%), *Helotiales_unclassified* (5.02%), *Pyrenochaeta* (2.39%), *Wickerhamomyces* (1.93%), *Pestalotiopsis* (1.48%), and *Trichosporon* (1.44%) were the dominant genera (>1%) in group B; *Ascomycota_unclassified* (14.20%), *Fungi_unclassified* (7.44%), *Davidiella* (5.72%), *Sordariomycetes_unclassified* (4.73%), *Zygomycota_unclassified* (3.46%), *Candida* (2.50%), *Mortierella* (2.48%), *Mucorales_unclassified* (1.90%), *Arthrinium* (1.65%), *Wickerhamomyces* (1.46%), *Helotiales_unclassified* (1.42%), *Pestalotiopsis* (1.29), *Gibberella* (1.20%), and *Preussia* (1.04%) were the dominant genera (>1%) in group C (Figure 4II).

### 3.4 Fungal isolation and identification

After the culturing of fungi in the fecal samples, 10 fungi, namely *Arthrinium* sp., *Trichoderma pubescens*, *Trichoderma* sp., *Pestalotiopsis* sp., *Mucor hiemalis*, *Didymella* sp., *Phoma* sp., *Simplicillium* sp., *Nectria pseudotrichia*, and *Bifusisporella* sp. were identified. Community composition analysis revealed that *Trichosporon* and *Cryptococcus* were not isolated. Supplementary Figure S4 shows the phylogenetic tree of fungi based on LEfSe analysis.

### 3.5 LEfSe analysis

**Figure 5:**
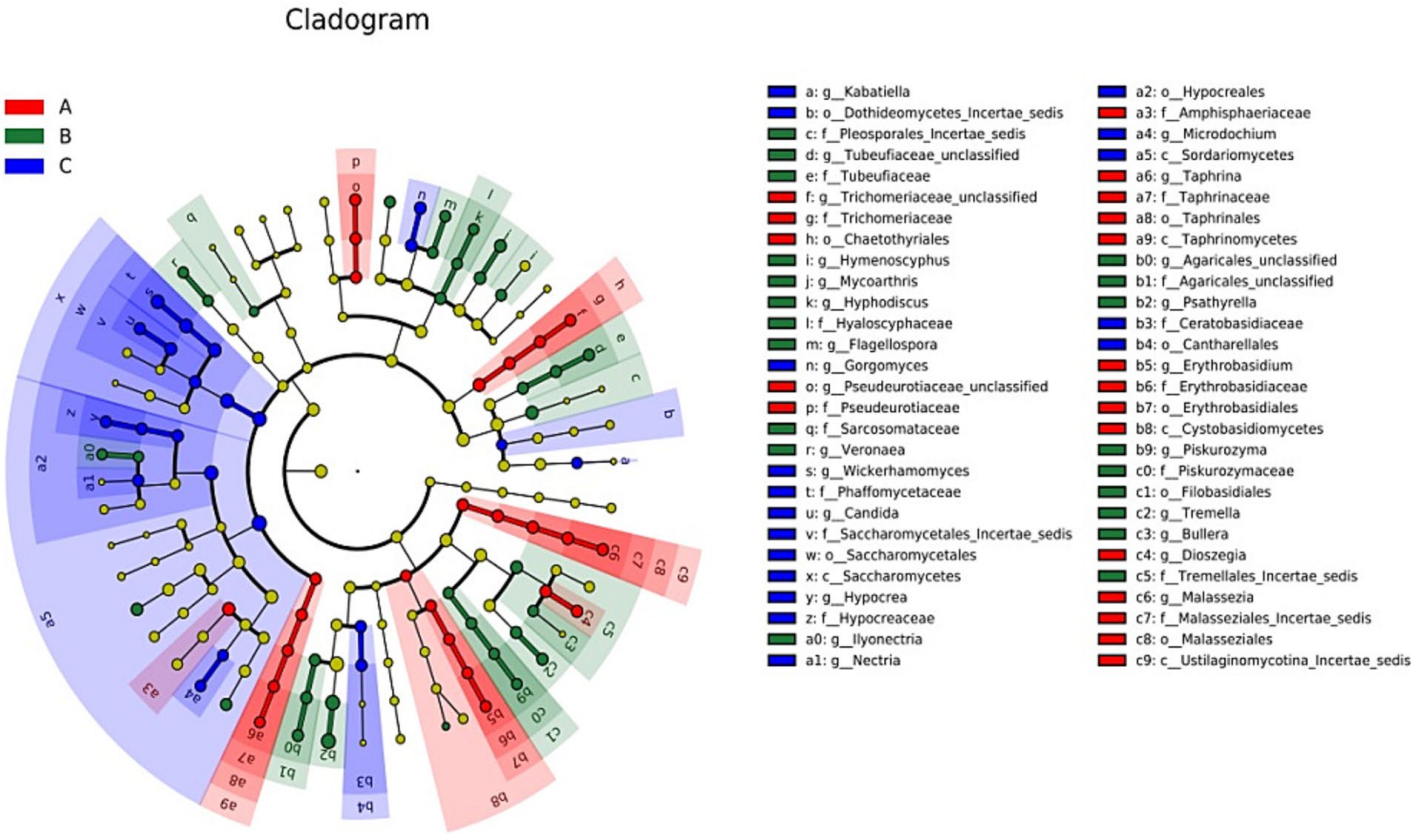
Linear discriminant analysis effect size (LEfSe) analysis results for the three groups based on the internal transcribed spacer rRNA gene. The different colors represent different groups. From the inside to the outside, phylum, class, order, family, and genus are shown. LEfSe analysis was primarily performed to compare several groups to identify species with significant differences in abundance between groups. In the cladogram, red, green, and blue represent group A and the circle represents the species level boundary from the inside to the outside. The yellow node indicates no significant difference; however, nodes shown in the color corresponding to a particular group indicate that the species has the highest abundance in that group.

**Figure 6:**
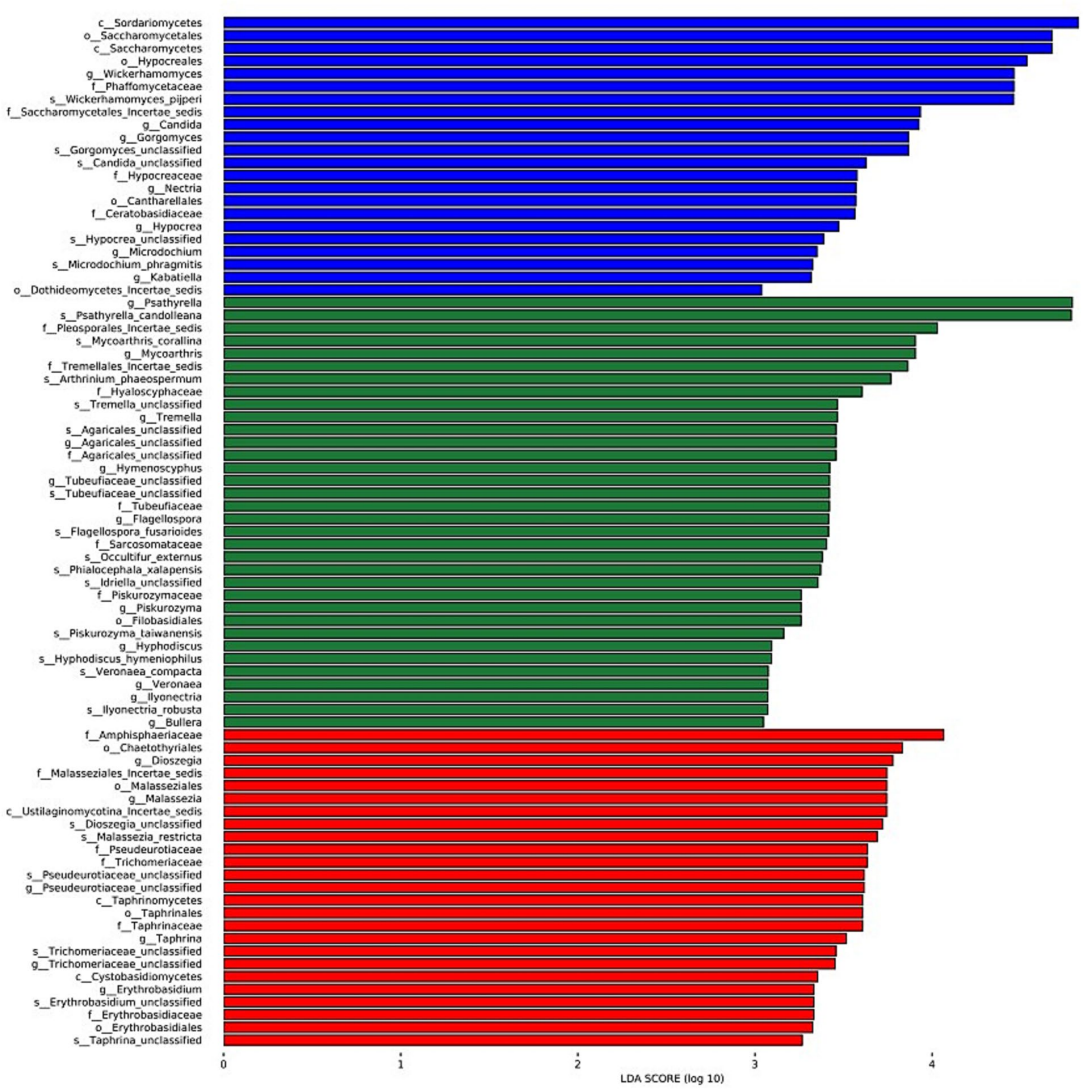
Linear discriminant analysis (LDA) results showing the distinct fungal genera in the three groups. The color of the bar chart represents the abundance of different species in each group, and the length represents the degree of significance (*p* < 0.05).

The LDA charts (Figures 5, 6) obtained after LEfSe analysis revealed no significant differences among the groups at the phylum level (*p* > 0.05). However, we observed significant differences at the genus level (*p* < 0.05) for *Wickerhamomyces*, *Candida*, *Gorgomyces*, *Nectria*, *Microdochium*, *Kabatiella,* and *Hypocrea* in group A; *Psathyrella*, *Mycoarthris*, *Tremella*, *Agaricales_unclassified*, *Hymenoscyphus*, *Tubeufiaceae_unclassified*, *Flagellospora*, *Piskurozyma*, *Hyphodiscus*, *Veronaea*, *Ilyonectria,* and *Bullera* in group B; and *Dioszegia*, *Malassezia*, *Pseudeurotiaceae_unclassified*, *Taphrina*, *Trichomeriaceae_unclassified*, and *Erythrobasidium* in group C.

### 3.6 Identification potentially pathogenic fungi genera

**Figure 7:**
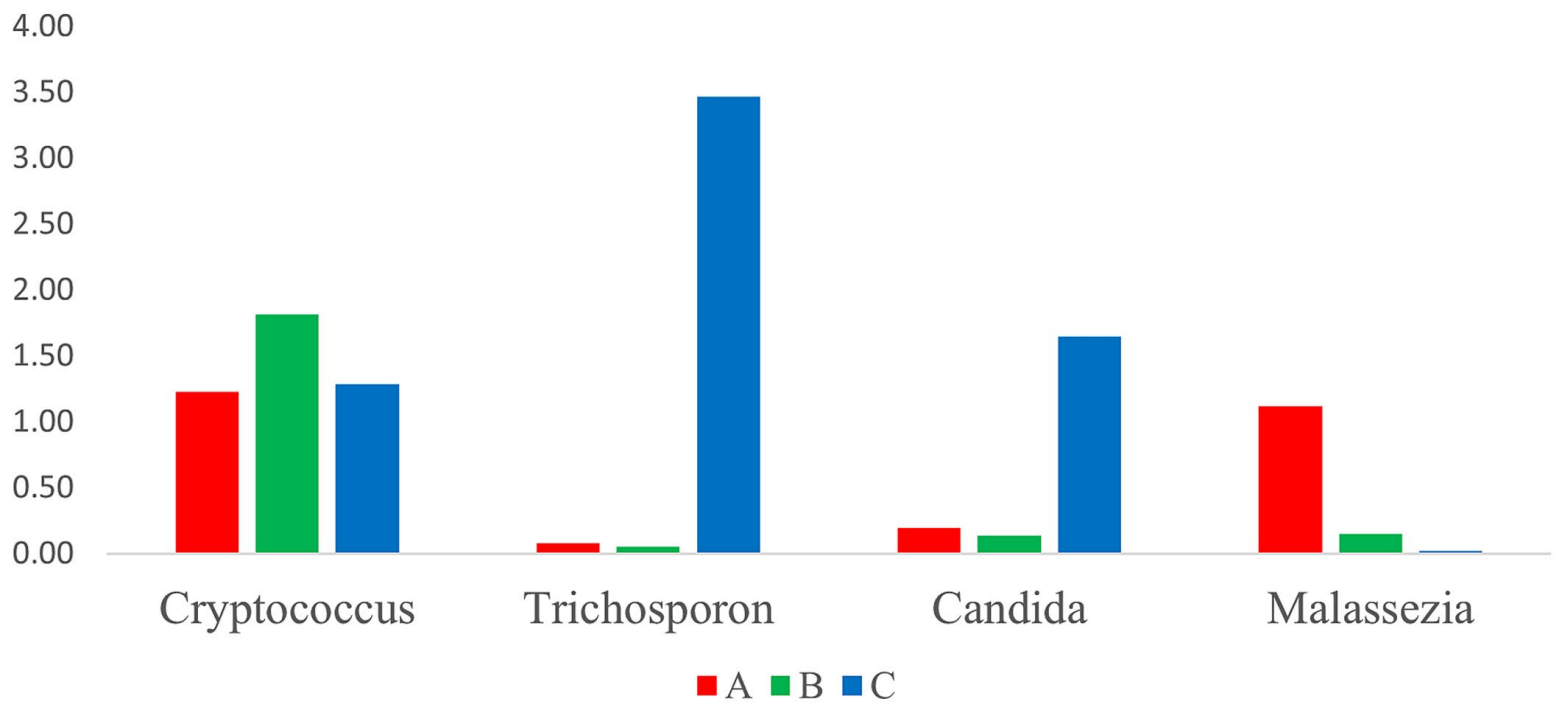
Comparative analysis of pathogenic fungi in the three groups.

Based on the determination of the relative abundances of potentially pathogenic fungi genera, such as *Cryptococcus*, *Trichosporon*, *Candida,* and *Malassezia*, we identified *Cryptococcus* as the most abundant genera, followed by *Trichosporon*, *Candida*, and *Malassezia* (Figure 7), and their relative abundances did not differ among the three groups. Further, the relative abundances of *Trichosporon* and *Candida* in group B were relatively high, and in group A, that of *Malassezia* was the highest.

## 4 Discussion

*A. rufipectus*, a key protected wild bird in China, is not differentiated by subspecies and is only distributed in a few counties in central Sichuan. To protect it from extinction, Sichuan set up a reserve to protect its habitat and performed rescue and self-breeding operations to expand its population. However, in establishing these measures, the effects of various microorganisms on *A. rufipectus* were not considered. Therefore, in this study, we employed high-throughput sequencing and traditional culture methods to analyze its fungal composition.

Estimated *α* and *β* diversity indices are reflective of diversity or heterogeneity in biological communities. The short distances and minimal differences between the three experimental groups based on the obtained α and β diversity indices could be attributed to the small interspecific differences between *A. rufipectus* and *L. nycthemera*, the short time interval between sampling (3 months), and the relatively small proportion of fungi in the intestinal tracts of the birds. Additionally, the similarities in intestinal tract environment, diet, and habitat may have also contributed to the slight differences observed among the three sample groups.

We examined the fungal composition of the gut of *A. rufipectus* without culturing via high-throughput ITS rRNA sequencing. The results obtained were consistent with previously reported core gut microbial communities in wild birds (26–28). Further, the predominance of three fungal taxa, Ascomycota, Basidiomycota, and Zygomycota, in the gut of *A. rufipectus* indicated that these fungi possibly play key roles in immune response, metabolism, and nutrient absorption of this species. A large proportion of fungi belonging to phylum Ascomycota reproduce asexually via spores and show rapid growth (29–31). Further, several Basidiomycota have been reported, and most of them are edible. Field environments also provide optimal conditions for Basidiomycota growth; therefore, they are highly abundant in nature. Therefore, these species may potentially become a primary food source for wild animals (29, 31).

In this study, we identified a total of 20 dominant genera. Excluding unclassified fungi, the intestinal fungi of wild *A. rufipectus* and *L. nycthemera* predominantly consisted of yeasts and some plant fungi, which may be related to the diet of the birds. Additionally, certain potential pathogenic fungi, including *Cryptococcus* spp., *Trichosporon* spp., *Candida* spp., and *Malassezia* spp., were identified. As described in section 3.6, the dominant genera in group C were *Trichosporon* spp. and *Candida* spp., while that in group A was *Malassezia* spp. Further, the relative abundance of *Cryptococcus* spp. was not significantly different among the three groups; however, it was more prevalent in summer. Therefore, *Trichosporon* spp. and *Candida* spp. may be associated with autumn, *Cryptococcus* spp. may be associated with summer, and *Malassezia* spp. may be related to *A. rufipectus*.

*Cryptococcus* spp. is an opportunistic pathogen that usually infects immunocompromised patients and invades the body via the bloodstream, and thereafter, reaches various organs (32). It is present in soil, bird droppings, and moist environments (33, 34), and previous studies have reported its impact on the human body. For example, it has been associated with pneumonia as well as central nervous system diseases. However, its effect on birds requires further exploration (35, 36). Recently, *Trichosporon* spp. was identified as class of invasive fungi that are widely distributed in nature, including air, soil, and wood (37–39). It has also been shown that they can colonize the human digestive and respiratory tracts (40, 41), causing superficial infections (42, 43). They can also cause invasive infections in humans, leading to fungemia and fungal pneumonia when immune function is weak (44–46). However, further research is required to ascertain the pathogenicity of *Trichosporon* spp. in birds. Our laboratory previously demonstrated that *Trichosporon* spp. can cause skin and liver damage in mice (42). *Candida* spp. is generally present in the natural environment and animals and can infect the skin and mucous membranes as well as the internal organs of animals (47). It has also been shown that they can colonize the skin, oral cavity, and digestive tract of some uninfected animals (48). Studies on candidiasis in birds have been primarily focused on broiler chickens. *Candida* spp. has a strong ability to adapt to its environment and can survive and multiply in the environment and body for a long time. Thus, its infection can reduce production performance and immunity in broiler birds, and also lead to other diseases (49–52). *Malassezia* spp. mainly colonizes the skin (53, 54); however, it has been reported that they can also colonize the intestinal tract (55–58) and are associated with the occurrence and development of inflammatory bowel disease, ulcerative colitis, irritable bowel syndrome, and Crohn’s disease (59–62). However, these studies were primarily focused on humans. Thus, the effects of these pathogens on birds require further investigation.

In this study, LEfSe analysis revealed the presence of numerous macrofungi in the gut of *A. rufipectus* in summer, including *Psathyrella* and *Tremella*, which differed from the findings obtained for *L. nycthemera* in summer and *A. rufipectus* in autumn. No macrofungi were observed in the gut of *L. nycthemera* in summer, while some macrofungi were observed in the gut of *A. rufipectus* in autumn. These findings can be explained by the heavy precipitation and humidity that characterize the summer period, and serve as optimal conditions for the growth of macrofungi. No macrofungi were observed in *L. nycthemera*, indicating that *A. rufipectus* may feed on macrofungi, while the former does not. Therefore, we reasoned that the two species exhibit slightly different eating habits.

An impaired immune system allows potential disease-causing fungi to grow rapidly and cause disease (63). In this study, we observed disease-causing fungi in all three groups, indicating that wild birds in the reserve carry opportunistic pathogens. In some cases, particularly when the immune system is compromised due to diverse stress factors, such as changes in environmental conditions, there is an increased risk of infection in different bird species (64). However, the observation of microorganisms in feces is not an accurate indication of the health status of an animal. For example, some pathogens can cause disease in other animals but not in birds. Given that we were unable to legally capture these birds for further research and verification, targeted etiological research was not possible. Regardless, our results provide a reference for the conservation of wild birds, such as *A. rufipectus*.

Fungal contamination or infection is closely related to wildlife health and conservation. On the one hand, it can cause disease, affect wildlife reproduction, and thus reduce wildlife populations; on the other hand, it can impair ecosystem functions (65). At the same time, the concept of “one health” is becoming more widely recognized. In the broader context of ecosystem health, human and wildlife health are closely linked (66, 67). Notably, anthropogenic pollution is also a threat to wildlife (68–70) e.g., pollutants from human activities may alter environmental conditions to promote fungal growth and reproduction, making these fungi more susceptible to infecting wildlife. Summarily, the protection of wildlife requires us to minimize man-made pollution, maintain ecological balance and provide a healthy living environment for wildlife.

In conclusion, we examined gut fungi in *A. rufipectus* via high-throughput ITS rRNA sequencing and traditional fungal culturing and used *L. nycthemera* for comparisons. Our results showed that these two bird species are similar in terms of the diversity, composition, and function of fungi in their guts. These findings may serve as a valuable reference for clarifying the biological habits of *A. rufipectus*, including its dietary habits. Further, several potential pathogenic fungi, such as *Cryptococcus* spp., *Trichosporon* spp., *Candida* spp., and *Malassezia* spp., which could serve as early warning signals for the protection of these valuable birds, were identified. Even though our study did not involve targeted etiological research to identify the etiopathogenesis of these potential pathogenic agents and we were unable to lawfully capture the birds for further research and verification, this study is the first report on gut fungi composition in wild *A. rufipectus* and provides a reference for the scientific conservation of this species. In future, it would be necessary to evaluate *A. rufipectus* samples obtained over a wider region and longer time period for more detailed comparisons.

## Data Availability

The datasets presented in this study can be found in online repositories. The names of the repository/repositories and accession number(s) can be found in the article/Supplementary material.

## Footnotes

^1^ https://www.omicstudio.cn/tool/

^2^ https://www.omicstudio.cn/analysis/
