# Supplementary material for: Analysis of fungal diversity in the feces of Arborophila rufipectus
Source: Front Vet Sci. 2024 Oct 14;11:1430518. doi: 10.3389/fvets.2024.1430518 (PMC11514364; doi:10.3389/fvets.2024.1430518)
Supplement: Supplementary file 1 [file Data_Sheet_1.pdf]

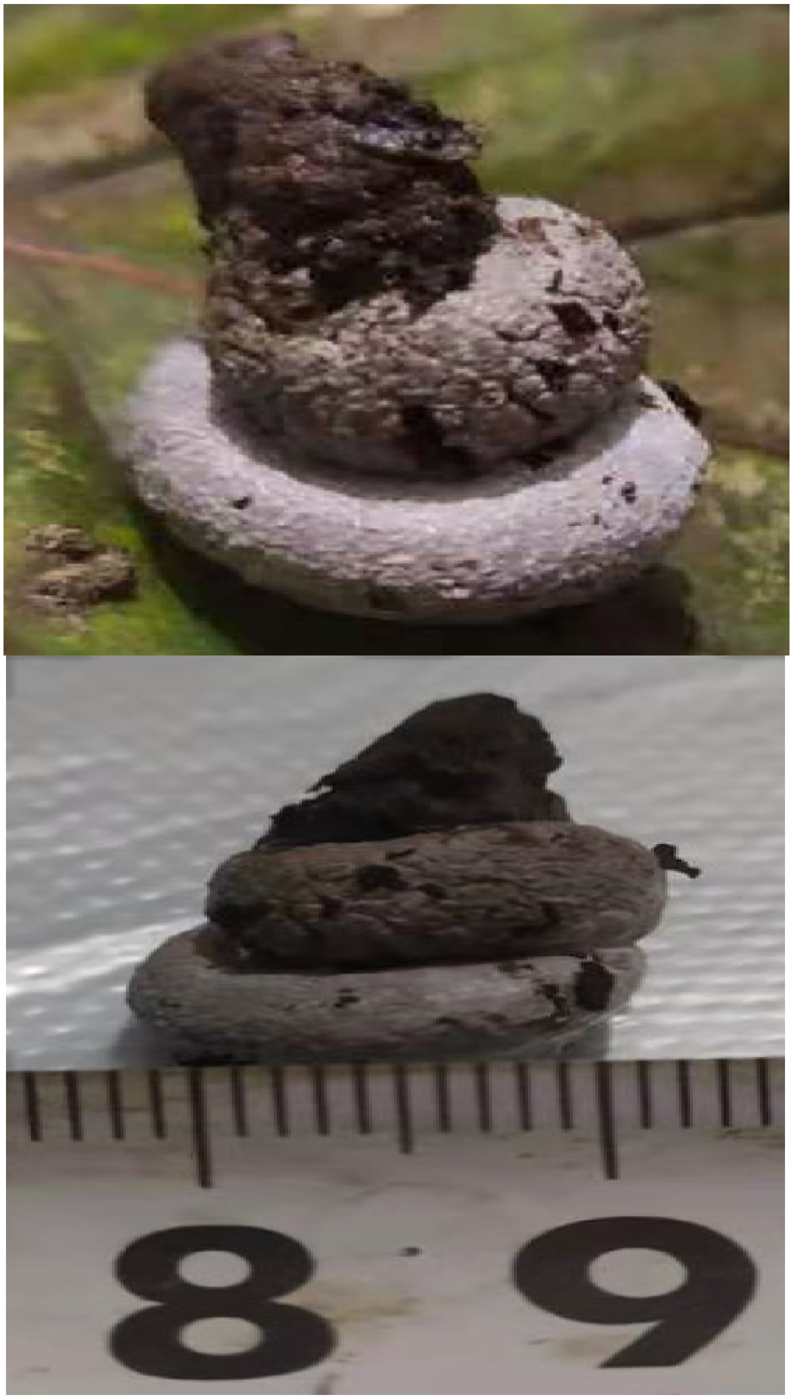


Figure S1.Morphological characteristics of feces of *A. rufipectus*

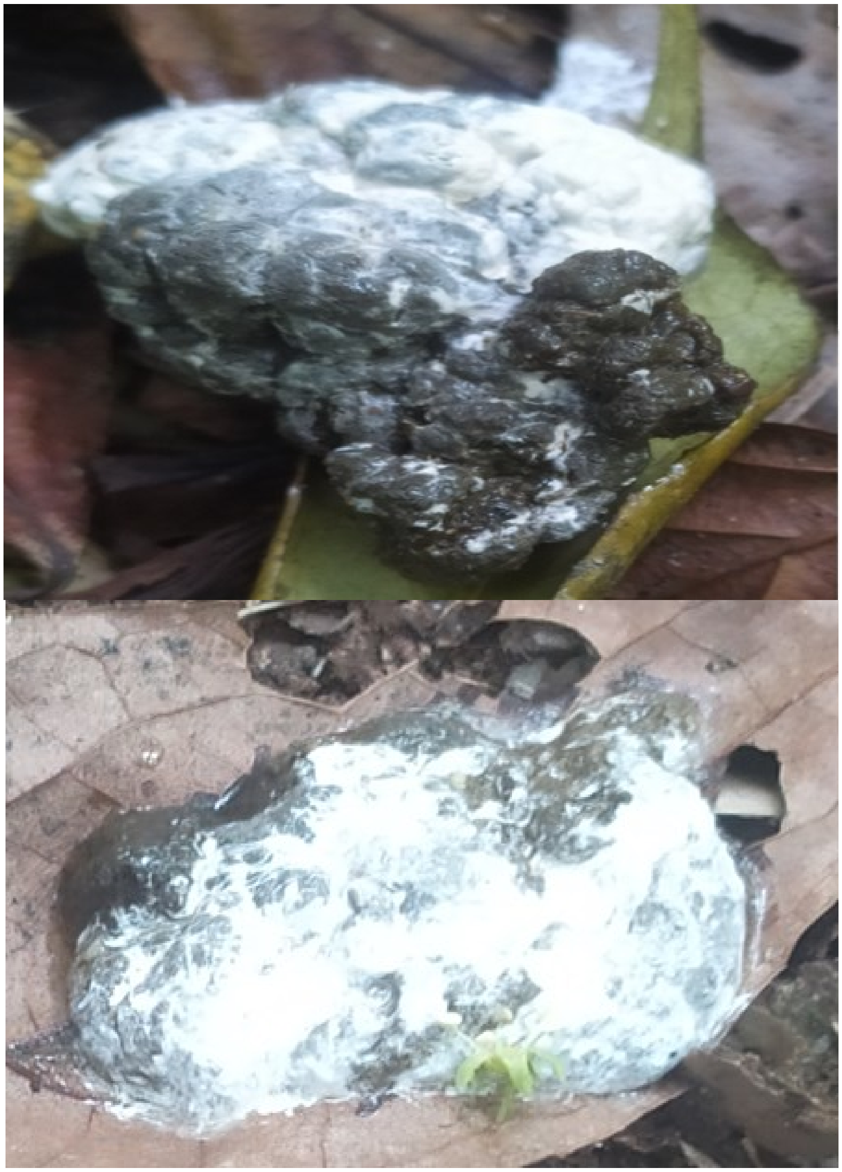

Figure S2. Morphological characteristics of feces of *L. nycthemera*
