# Supplementary figures and images for: Analysis of fungal diversity in the feces of Arborophila rufipectus
Source: Front Vet Sci. 2024 Oct 14;11:1430518. doi: 10.3389/fvets.2024.1430518 (PMC11514364; doi:10.3389/fvets.2024.1430518)

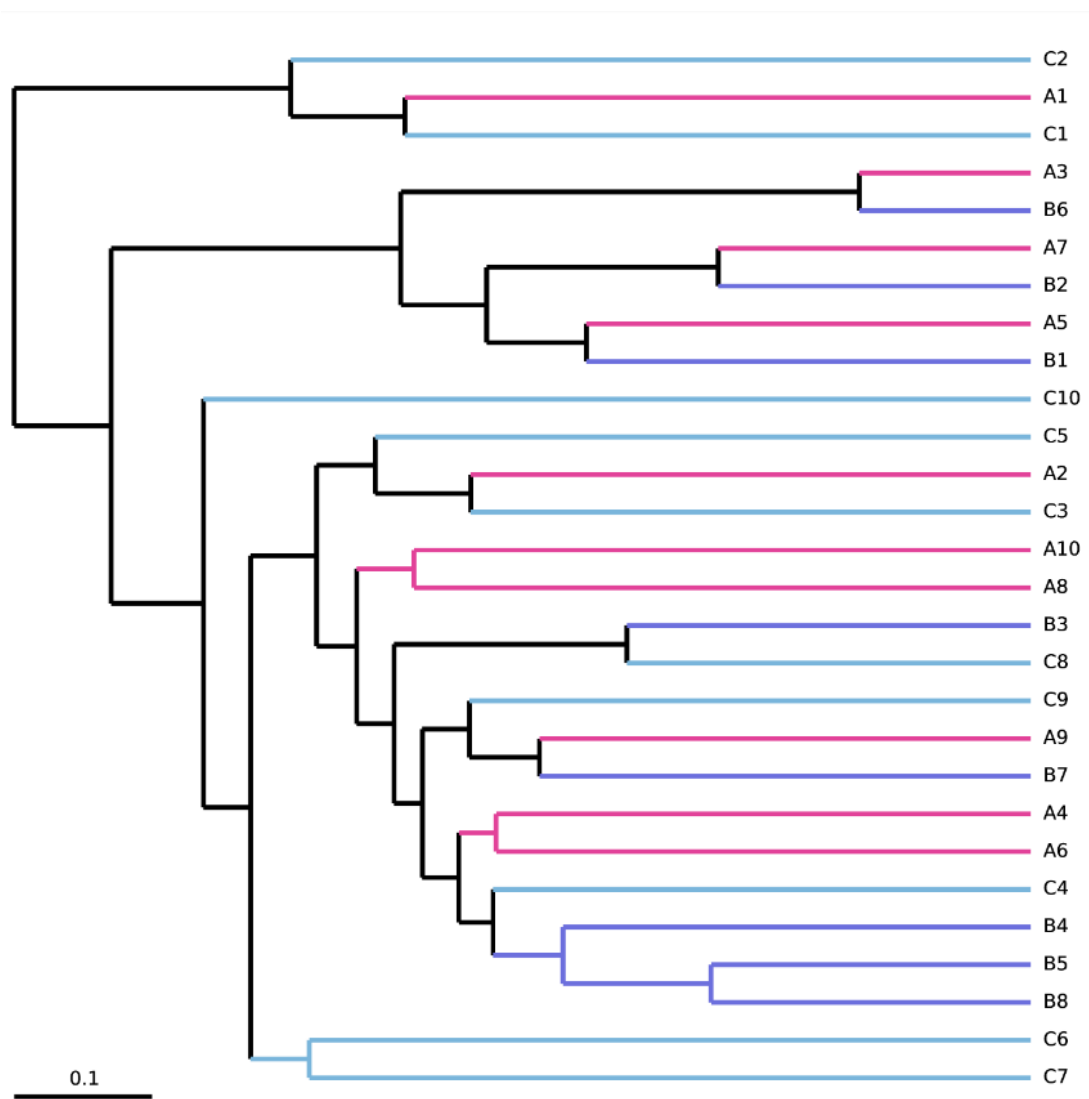

**Fig S3** unweighted uniFrac UPGMA cluster

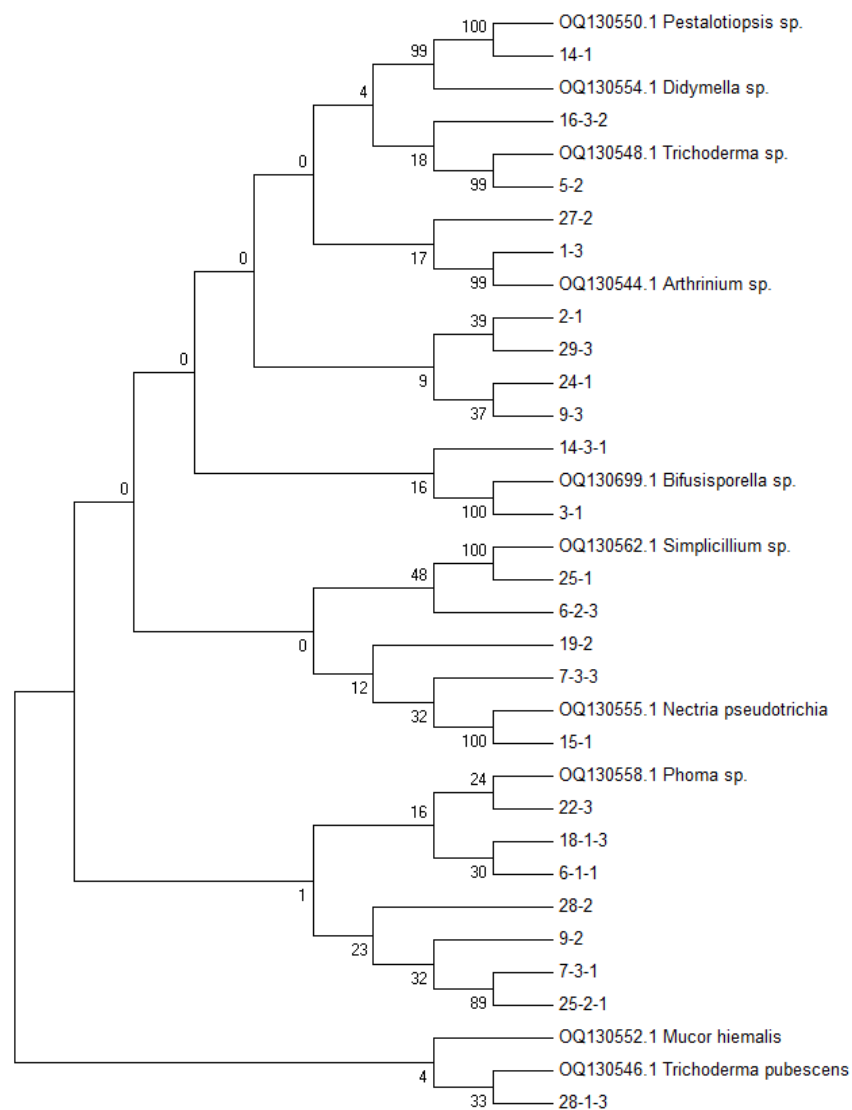

**Fig S4** Dendrogram of isolated and identified fung

Supplement: Supplementary file 2 [file Data_Sheet_2.pdf]
