# Supplementary material for: Analysis of fungal diversity in the feces of Arborophila rufipectus
Source: Front Vet Sci. 2024 Oct 14;11:1430518. doi: 10.3389/fvets.2024.1430518 (PMC11514364; doi:10.3389/fvets.2024.1430518)
Supplement: Supplementary file 3 [file Data_Sheet_3.pdf]

Sampling table of each group

Samples	Animal	Longitude and latitude	Time
A1	Lophura Nycthemera	N 28°42′ 00″ E 104°23′ 36″	2021/6/19
A2	Lophura Nycthemera	N 28°41′ 30″ E 104°15′ 07″	2021/6/19
A3	Lophura Nycthemera	N 28°42′ 00″ E 104°05′ 30″	2021/6/20
A4	Lophura Nycthemera	N 28°41′ 59″ E 104°05′ 07″	2021/6/20
A5	Lophura Nycthemera	N 28°41′ 56″ E 104°05′ 09″	2021/6/20
A6	Lophura Nycthemera	N 28°42′ 00″ E 104°05′ 55″	2021/6/20
A7	Lophura Nycthemera	N 28°42′ 22″ E 104°21′ 02″	2021/6/21
A8	Lophura Nycthemera	N 28°42′ 03″ E 104°24′ 06″	2021/6/24
A9	Lophura Nycthemera	N 28°42′ 01″ E 104°21′ 11″	2021/6/27
A10	Lophura Nycthemera	N 28°42′ 00″ E 104°01′ 51″	2021/7/1
B1	Arborophila Rufipectus	N 28°46′ 38″ E 103°59′ 38″	2021/6/19
B2	Arborophila Rufipectus	N 28°46′ 38″ E 103°59′ 38″	2021/6/19
B3	Arborophila Rufipectus	N 28°44′ 16″ E 104°02′ 11″	2021/6/19
B4	Arborophila Rufipectus	N 28°46′ 38″ E 104°59′ 09″	2021/6/20
B5	Arborophila Rufipectus	N 28°42′ 44″ E 104°24′ 00″	2021/6/22
B6	Arborophila Rufipectus	N 28°42′ 46″ E 104°21′ 01″	2021/6/22
B7	Arborophila Rufipectus	N 28°42′ 02″ E 104°21′ 12″	2021/6/24
B8	Arborophila Rufipectus	N 28°42′ 01″ E 104°28′ 13″	2021/6/26
B9	Arborophila Rufipectus	N 28°42′ 15″ E 104°15′ 08″	2021/6/28
B10	Arborophila Rufipectus	N 28°42′ 00″ E 104°15′ 05″	2021/6/28
C1	Arborophila Rufipectus	N 28°42′ 21″ E 104°42′ 00″	2021/9/7
C2	Arborophila Rufipectus	N 28°44′ 37″ E 104°05′ 09″	2021/9/9
C3	Arborophila Rufipectus	N 28°42′ 57″ E 104°21′ 08″	2021/9/9
C4	Arborophila Rufipectus	N 28°41′ 53″ E 104°22′ 26″	2021/9/10
C5	Arborophila Rufipectus	N 28°41′ 55″ E 104°01′ 19″	2021/9/12
C6	Arborophila Rufipectus	N 28°42′ 00″ E 104°02′ 07″	2021/9/15
C7	Arborophila Rufipectus	N 28°41′ 49″ E 104°02′ 34″	2021/9/15
C8	Arborophila Rufipectus	N 28°41′ 56″ E 104°01′ 14″	2021/9/21
C9	Arborophila Rufipectus	N 28°42′ 00″ E 104°01′ 57″	2021/9/22
C10	Arborophila Rufipectus	N 28°42′ 03″ E 104°02′ 03″	2021/9/23

Alpha diversity index of each sample（ITS rRNA）

samples	shannon	simpson	chao1	goods_coverage
A1	4.37	0.75	397.07	1.00
A2	5.71	0.94	323.00	1.00
A3	4.46	0.89	193.00	1.00
A4	2.70	0.50	349.40	1.00
A5	5.20	0.95	141.00	1.00
A6	5.08	0.92	179.00	1.00
A7	6.03	0.97	204.00	1.00
A8	1.62	0.27	280.40	1.00
A9	4.49	0.89	137.00	1.00
A10	5.84	0.97	197.00	1.00
B1	4.23	0.79	200.00	1.00
B2	4.70	0.90	223.27	1.00
B3	4.63	0.87	313.00	1.00
B4	6.44	0.98	248.00	1.00
B5	6.82	0.98	336.17	1.00
B6	2.00	0.43	174.55	1.00
B7	6.41	0.98	262.00	1.00
B8	6.26	0.95	492.23	1.00
B9	3.31	0.62	138.17	1.00
B10	3.26	0.73	283.45	1.00
C1	4.62	0.83	134.50	1.00
C2	2.40	0.55	158.09	1.00
C3	5.99	0.96	372.05	1.00
C4	6.44	0.98	352.26	1.00
C5	5.08	0.93	232.00	1.00
C6	2.93	0.58	335.36	1.00
C7	3.76	0.79	400.41	1.00
C8	5.58	0.95	142.00	1.00
C9	2.44	0.57	270.76	1.00
C10	5.41	0.94	164.00	1.00

Blastn alignments and nucleotide sequence accession numb

Sample	ITS sequence comparison	ITS GenBank serial number
1-3	Arthrinium sp.	OQ130544
2-1	Arthrinium sp.	OQ130545
18-1-3	Trichoderma pubescens	OQ130546
9-2	Trichoderma pubescens	OQ130547
5-2	Trichoderma sp.	OQ130548
24-1	Trichoderma sp.	OQ130549
22-3	Trichoderma sp.	OQ130560
28-1-3	Trichoderma sp.	OQ130561
14-1	Pestalotiopsis sp.	OQ130550
29-3	Pestalotiopsis sp.	OQ130551
7-3-1	Mucor hiemalis	OQ130552
7-3-3	Mucor hiemalis	OQ130553
9-3	Didymella sp.	OQ130554
27-2	Didymella sp.	OQ130557
6-2-3	Phoma sp.	OQ130558
6-1-1	Phoma sp.	OQ130559
25-1	Simplicillium sp.	OQ130562
25-2-1	Simplicillium sp.	OQ130563
15-1	Nectria pseudotrichia	OQ130555
19-2	Nectria pseudotrichia	OQ130556
3-1	Bifusisporella sp.	OQ130699
